# Vegetarian diet improves insulin resistance and oxidative stress markers more than conventional diet in subjects with Type 2 diabetes

**DOI:** 10.1111/j.1464-5491.2010.03209.x

**Published:** 2011-05

**Authors:** H Kahleova, M Matoulek, H Malinska, O Oliyarnik, L Kazdova, T Neskudla, A Skoch, M Hajek, M Hill, M Kahle, T Pelikanova

**Affiliations:** * Institute for Clinical and Experimental Medicine, Charles University1st Faculty of Medicine, Prague, Czech Republic; † Institute of EndocrinologyPrague, Czech Republic

**Keywords:** exercise, insulin resistance, oxidative stress markers, vegetarian diet, visceral fat

## Abstract

**Aims:**

The aim of this study was to compare the effects of calorie-restricted vegetarian and conventional diabetic diets alone and in combination with exercise on insulin resistance, visceral fat and oxidative stress markers in subjects with Type 2 diabetes.

**Methods:**

A 24-week, randomized, open, parallel design was used. Seventy-four patients with Type 2 diabetes were randomly assigned to either the experimental group (*n* = 37), which received a vegetarian diet, or the control group (*n* = 37), which received a conventional diabetic diet. Both diets were isocaloric, calorie restricted (-500 kcal/day). All meals during the study were provided. The second 12 weeks of the diet were combined with aerobic exercise. Participants were examined at baseline, 12 weeks and 24 weeks. Primary outcomes were: insulin sensitivity measured by hyperinsulinaemic isoglycaemic clamp; volume of visceral and subcutaneous fat measured by magnetic resonance imaging; and oxidative stress measured by thiobarbituric acid reactive substances. Analyses were by intention to treat.

**Results:**

Forty-three per cent of participants in the experimental group and 5% of participants in the control group reduced diabetes medication (*P* < 0.001). Body weight decreased more in the experimental group than in the control group [–6.2 kg (95% CI –6.6 to –5.3) vs. –3.2 kg (95% CI –3.7 to –2.5); interaction group × time *P* = 0.001]. An increase in insulin sensitivity was significantly greater in the experimental group than in the control group [30% (95% CI 24.5–39) vs. 20% (95% CI 14–25), *P* = 0.04]. A reduction in both visceral and subcutaneous fat was greater in the experimental group than in the control group (*P* = 0.007 and *P* = 0.02, respectively). Plasma adiponectin increased (*P* = 0.02) and leptin decreased (*P* = 0.02) in the experimental group, with no change in the control group. Vitamin C, superoxide dismutase and reduced glutathione increased in the experimental group (*P* = 0.002, *P* < 0.001 and *P* = 0.02, respectively). Differences between groups were greater after the addition of exercise training. Changes in insulin sensitivity and enzymatic oxidative stress markers correlated with changes in visceral fat.

**Conclusions:**

A calorie-restricted vegetarian diet had greater capacity to improve insulin sensitivity compared with a conventional diabetic diet over 24 weeks. The greater loss of visceral fat and improvements in plasma concentrations of adipokines and oxidative stress markers with this diet may be responsible for the reduction of insulin resistance. The addition of exercise training further augmented the improved outcomes with the vegetarian diet.

## Introduction

Type 2 diabetes is only half as prevalent in vegetarians compared with non-vegetarians [1,2]. Randomized controlled intervention studies in patients with Type 2 diabetes have shown greater weight loss, reduction in fasting plasma glucose [3], greater improvements in HbA_1c_ and fasting and postprandial lipids [4,5], and reduction of diabetes medications [3–5] with vegetarian diets compared with more conventional diets used to treat diabetes. The mechanisms have not been fully elucidated.

Exercise training reduces insulin resistance by a number of mechanisms: preferential loss of visceral fat, stimulation of muscle development, increased skeletal muscle insulin action, morphological changes in muscle and improved control over hepatic glucose production [6]. To our knowledge, the effects of the combination of a vegetarian diet and exercise training compared with conventional diets in combination with exercise training on insulin resistance, resting energy expenditure and volume of visceral fat in patients with Type 2 diabetes have not yet been studied.

The aim of our study was to compare the effects of isocaloric, calorie-restricted vegetarian and conventional diabetic diets on insulin resistance, volume of visceral fat and plasma markers of oxidative stress after a 3-month dietary-intervention phase and to test whether the positive changes will be sustainable or even augmented after adding aerobic exercise training for an additional 3 months. Our hypothesis was that a vegetarian diet would be more effective in reducing insulin resistance and volume of visceral fat and improving oxidative stress markers than a conventional diabetic diet and there would be a further enhancement of the difference between groups after the addition of exercise training.

## Patients and methods

### Subjects

**FIGURE 1:**
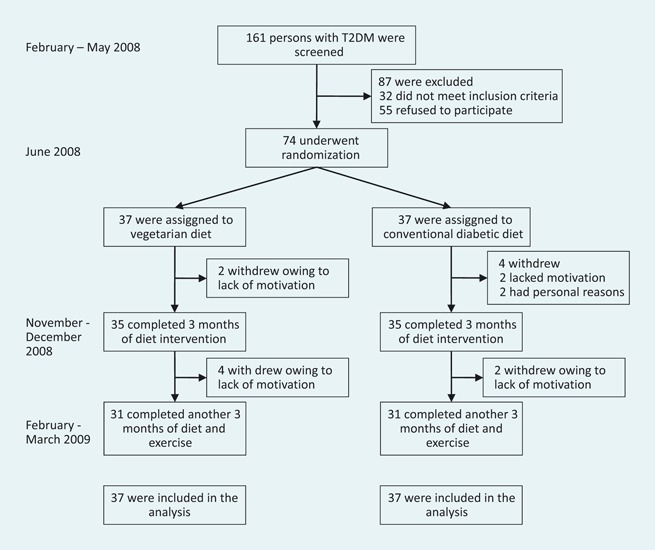
Enrollment of the participants and completion of the study. T2DM, Type 2 diabetes mellitus.

Subjects with Type 2 diabetes treated by oral hypoglycaemic agents were recruited from February to May 2008. Inclusion criteria were: Type 2 diabetes, age 30–70 years, HbA_1c_ between 6 and 11% (42–97 mmol/mol), BMI between 25 and 53 kg/m^2^, and willingness to change dietary habits and follow a prescribed exercise programme. Exclusion criteria were HbA_1c_ < 6% (< 42 mmol/mol) or > 11% (> 97 mmol/mol), use of insulin, abuse of alcohol or drugs, pregnancy, lactation, or current use of a vegetarian diet. Out of 161 patients pre-chosen by their endocrinologists, 74 met the inclusion criteria and gave written informed consent (Fig. 1, Table 1).

**Table 1:** Baseline characteristics of the study population

Characteristic	Experimental group (*n* = 37)	Control group (*n* = 37)
Age (years)	54.6 ± 7.8	57.7 ± 4.9
Sex (%)		
Male	17 (46)	18 (49)
Female	20 (54)	19 (51)
Smokers (%)	9 (24)	5 (14)
Weight (kg)	101.1 ± 17.1	100.8 ± 17.8
BMI (kg m^−2^)	35.1 ± 6.1	35.0 ± 4.6
Waist circumference (cm)	113.7 ± 11.2	113.8 ± 13.1
Blood pressure (mmHg)		
Systolic	133 ± 16	130 ± 13
Diastolic	85 ± 13	84 ± 8
Resting heart rate (beats min^−1^)	73 ± 7	74 ± 10
Volume of abdominal fat (ml)		
Visceral fat	4266 ± 1994	4275 ± 1954
Subcutaneous fat	8140 ± 3847	8110 ± 2739
Metabolic clearance rate of glucose (ml kg^−1^ min^−1^)	2.29 ± 0.8	2.24 ± 0.8
Blood biomarkers		
Cholesterol total (mmol l^−1^)	4.4 ± 0.8	4.2 ± 0.9
LDL cholesterol (mmol l^−1^)	2.54 ± 0.6	2.57 ± 0.8
HDL cholesterol (mmol l^−1^)	1.07 ± 0.3	1.09 ± 0.2
Triglycerides (mmol l^−1^)	2.1 ± 0.9	2.1 ± 0.9
Homocysteine (μmol l^−1^)	12.5 ± 4.5	13.1 ± 3.7
Free fatty acids (mmol l^−1^)	0.54 ± 0.3	0.58 ± 0.4
Fibrinogen (g l^−1^)	4.3 ± 1.3	3.9 ± 1.1
Fasting plasma immunoreactive insulin (nmol l^−1^)	15.4 ± 8.0	14.8 ± 9.3
Fasting plasma C-peptide (mIU l^−1^)	1.3 ± 0.6	1.4 ± 0.6
Fasting plasma glucose (mmol l^−1^)	9.5 ± 2.8	9.5 ± 2.4
HbA_1c_ [DCCT, %; (IFCC, mmol/mol)]	7.6 ± 1.4 (60 ± 14)	7.7 ± 1.2 (61 ± 12)
hsCRP (mg l^−1^)	5.6 ± 6.3	5.5 ± 4.8
Adiponectin total (μg ml^−1^)	6.7 ± 3.8	6.8 ± 3.1
HMW adiponectin (μg ml^−1^)	4.0 ± 2.0	4.0 ± 2.6
Leptin (pm)	1100 ± 1121	1112 ± 946
Resistin (ng ml^−1^)	9.47 ± 5.8	9.82 ± 4.6
Vitamin C (μg mmol^−1^)	80.7 ± 22.0	81.3 ± 24.2
Superoxide dismutase (U ml^−1^)	4.7 ± 2.2	4.7 ± 1.1
Catalase (H_2_O_2_.min^−1^ mg^−1^)	679 ± 189	659 ± 198
TBARS (μmol l^−1^)	1.7 ± 0.6	1.7 ± 0.4
Reduced glutathione (mmol l^−1^)	2.7 ± 0.9	2.7 ± 0.6
Glutathione reductase (mmol NADPH min^−1^ mg^−1^)	259 ± 97	262 ± 94
Glutathione peroxidase (mmol GSH min^−1^ mg^−1^)	631 ± 147	627 ± 125
Glutathione transferase (mmol GSH min^−1^ mg^−1^)	32.2 ± 11.5	34.1 ± 9.1
Medications		
Oral hypoglycaemic agents (%)		
Metformin	29 (78)	28 (76)
Sulphonylurea	20 (54)	13 (35)
Thiazolidinedione	7 (19)	5 (14)
Other	8 (22)	3 (8)
Lipid-lowering therapy (%)	22 (59)	16 (43)
Anti-hypertensive therapy (%)	25 (68)	22 (59)
Indirect calorimetry		
Respiratory quotient	0.77 ± 0.1	0.76 ± 0.1
Resting energy expenditure (REE) (kcal day^−1^)	1792 ± 317	1727 ± 322
REE (% of predicted value)	111.9 ± 49.4	106.5 ± 35
Fasting oxidation of carbohydrates (mg kg^−1^ min^−1^)	0.73 ± 0.66	0.73 ± 0.66
Fasting oxidation of fat (mg kg^−1^ min^−1^)	0.71 ± 0.25	0.72 ± 0.4
Fasting oxidation of protein (mg kg^−1^ min^−1^)	0.73 ± 0.33	0.73 ± 0.15
Dietary intake		
Caloric intake (kcal day^−1^)	1835 ± 473	1832 ± 655
Carbohydrates (% of daily energy)	41.7 ± 7.58	42.3 ± 8.23
Fats (% of daily energy)	37.94 ± 6.46	37.89 ± 6.53
Proteins (% of daily energy)	20.55 ± 4.66	18.42 ± 3.31
Fibre intake (g day^−1^)	22.87 ± 7.06	22.95 ± 6.51
P/S ratio	0.69 ± 0.28	0.53 ± 0.19
Cholesterol intake (mg day^−1^)	386 ± 181	381 ± 224
Quality of life		
OWLQOL score	44.85 ± 23.69	40 ± 20.4
WRSM score	32.29 ± 26.18	30.77 ± 18.16

Data are means ± sd.

DCCT, Diabetes Control and Complications Trial; GSH, glutathione; H_2_O_2_, hydrogen peroxide; HMW, high molecular weight; hsCRP, high-sensitivity C-reactive protein; IFCC, International Federation of Clinical Chemistry and Laboratory Medicine; NADPH, nicotinamide adenine dinucleotide phosphate; OWLQOL, Obesity and Weight-Loss Quality-of-Life; P/S ratio, ratio of polyunsaturated to saturated fatty acids; TBARS, thiobarbituric acid reactive substances; WRSM, Weight-Related Symptoms.

### Study design

A 24-week, randomized, open, parallel, metabolically controlled design was used. The subjects were randomly assigned to either the experimental group (*n* = 37), which received a vegetarian diet, or the control group (*n* = 37), which received a conventional diabetic diet. Both diets were designed to be isocaloric and calorie restricted (-500 kcal/day), with caloric intakes based on the measurement of resting energy expenditure of each subject by indirect calorimetry (metabolic monitor VMAX; Sensor Medics, Anaheim, CA, USA) [7]. The second 12 weeks of the diet were combined with aerobic exercise. All participants started with a 1-week tutorial, where they learned in detail how to compose and prepare their diet. Participants attended weekly 1-h meetings with lectures and cooking classes. All meals during the study were provided. Participants were examined at baseline, 12 weeks and 24 weeks. The study protocol was approved by the Institutional Ethics Committee.

### Diet

The vegetarian diet (∼60% of energy from carbohydrates, 15% protein and 25% fat) consisted of vegetables, grains, legumes, fruits and nuts. Animal products were limited to maximum of one portion of low-fat yogurt a day. The conventional diabetic diet was administered according to the dietary guidelines of the Diabetes and Nutrition Study Group (DNSG) of the European Association for the Study of Diabetes (EASD). It contained 50% of total energy from carbohydrates, 20% protein, less than 30% fat (≤ 7% saturated fat, < 200 mg/day of cholesterol/day).

Vegetarian meals were provided in two vegetarian restaurants and the conventional diabetic diet meals were provided at the Institute for Clinical and Experimental Medicine, Prague. To meet the vitamin B_12_ needs of the experimental group, while maintaining the same level of intervention in the two groups, vitamin B_12_ was supplemented in both the experimental group and the control group (50 μg/day). Alcoholic beverages were limited to one per day for women and two per day for men.

### Exercise programme

Participants were asked not to alter their exercise habits during the first 12 weeks. During weeks 13–24 they were prescribed an individualized exercise programme based on their history of physical activity and an initial spiroergometric examination. Participants exercised at 60% of their maximal heart rate twice a week for 1 h under professional supervision, plus once a week at home or at the sports centre with the same intensity; they were given a sport-tester Polar FT4 (Polar, Kempele, Finnland) and a pedometer (Omron HJ-113, Omron, Kyoto, Japan) for individual physical activities and were repeatedly instructed on how to use them.

### Compliance

Records of all visits to pick up meals were kept. At weeks 0, 12 and 24, a 3-day dietary record was completed by each participant (two weekdays and one weekend day). A registered dietician analysed all 3-day dietary records using a country-specific food-nutrient database [8]. At weeks 3, 8, 14 and 19, a registered dietician made unannounced telephone calls and each participant recalled his or her 24-h diet. This data set was not statistically analysed, but allowed the investigators to check the adherence and to provide additional counselling. Participants were divided according to their adherence to the prescribed diet into the high, medium or low adherence group. High adherence was defined as the average daily energy intake being no more than 100 kcal in excess of the intake prescribed; medium adherence was less than 200 kcal in excess. If criteria for neither high nor medium adherence were met, the participants were included in the low adherence group. An additional criterion for high adherence to the vegetarian diet was the average daily cholesterol intake being ≤ 50 mg and, for medium adherence, being less than 100 mg. In the control group, the average daily cholesterol limit was ≤ 200 mg for high adherence and less than 300 mg for medium adherence.

### Physical activity

Physical activity was assessed by pedometer Omron HJ-113 (Omron, Kyoto, Japan); each participant completed a 3-day record, two weekdays and one weekend day, and with two questionnaires: the International Physical Activity Questionnaire (IPAQ) [9] and the Baecke questionnaire [10] at weeks 0, 12 and 24. Records of each participant’s visits to the sports centre were kept. Adherence to the exercise programme was defined as more than 75% of prescribed visits to the centre (18/24).

### Quality of life

Quality of life was assessed using two questionnaires: Obesity and Weight-Loss Quality-of-Life (OWLQOL) and Weight-Related Symptoms (WRSM) [11].

### Medication

Participants were asked to continue their pre-existing medication regimens, except when hypoglycaemia occurred repeatedly (fasting plasma glucose determined at the laboratory < 4.4 mmol l^−1^ or capillary glucose reading < 3.4 mmol l^−1^ accompanied by hypoglycaemic symptoms). In such cases, medications were reduced by a study physician following the medication protocol. All participants were given an Accu-Chek Go glucometer (Roche, Basel, Switzerland) and were instructed on how to use it.

### Procedures

All measurements were performed at 0, 12 and 24 weeks on an outpatient basis, after 10- to 12-h overnight fasting with only tap water allowed *ad libitum*. Height and weight were measured using a periodically calibrated scale accurate to 0.1 kg. Waist circumference was measured with a tape measure placed at the midpoint between the lowest rib and the upper part of the iliac bone. Blood pressure and heart rate were measured after participants had rested in a seated position for 5 min using a digital M6 Comfort monitor (Omron, Kyoto, Japan). Three measurements were taken at 2-min intervals. The first measurement was disregarded and a mean value was calculated for the remaining two measurements.

#### Hyperinsulinaemic isoglycaemic clamp

The hyperinsulinaemic (1 mU kg^−1^ min^−1^) isoglycaemic clamp, lasting 3 h, was conducted as previously described [12]. Insulin sensitivity was estimated as the metabolic clearance rate of glucose (MCR) calculated during the last 20 min of the clamp after correction for changes in glucose pool size [12].

#### Magnetic resonance imaging

Twenty-seven water-suppressed magnetic resonance images centred to the intervertebral disc of L2/L3 with repetition time/echo time (TR/TE) = 450/10 ms and thickness of 10 mm were acquired during breath-hold. The post-processing of magnetic resonance images with the calculation of subcutaneous and visceral abdominal fat volume was carried out in MATLAB (The Math Works, Natick, MA, USA); the inner border of the subcutaneous region was detected semi-automatically [13], while the abdominal fat voxels were selected by thresholding.

### Analytical methods

Plasma glucose was analysed using the Beckman Analyzer glucose–oxidase method (Beckman Instruments Inc., Fullerton, CA, USA). Plasma immunoreactive insulin and C-peptide concentrations were determined using Insulin and C-peptide IRMA kits (Immunotech, Prague, Czech Republic). HbA_1c_ was measured by HPLC (Tosoh, Tokyo, Japan). Plasma lipid concentrations were measured by enzymatic methods (Roche). HDL cholesterol was measured after double precipitation with dextran and magnesium chloride. LDL cholesterol was estimated using the Friedewald equation if triglyceride concentration was within normal limits.

#### Oxidative stress markers

The amount of lipid peroxidation was determined as thiobarbituric acid reactive substances (TBARS) [14] using home-made kits. The activity of superoxide dismutase, catalase, seleno-dependent glutathione peroxidase and the level of ascorbic acid were analysed by standard methods [15,16] using the following kits: superoxide dismutase assay kit (Sigma-Aldrich, St Louis, MO, USA), catalase and glutathione peroxidase assay kits (Cayman Chemical, Ann Arbor, MI, USA) and home-made kits for ascorbic acid. Glutathione reductase activity was measured by the decrease of NADPH (Sigma-Aldrich). The whole blood level of reduced glutathione was determined by the Glutathione HPLC Kit in whole blood (Chromsystems, Munich, Germany).

#### Adipokines

Plasma concentrations of total adiponectin and resistin were measured using ELISA kits (Raybiotech, Norcross, GA, USA), high molecular weight (HMW) adiponectin using ELISA kits (Millipore, Billerica, MA, USA) and leptin using Milliplex (Millipore).

### Statistical analyses

The intention-to-treat analysis included all participants. Repeated-measures ANOVA models with between-subject and within-subject factors and interactions were used for evaluation of the relationships between continuous variables and factors. Factors groups, subject and time, were included in the model. Interactions between group and time (group × time) were calculated for each variable. Within each group, paired comparison *t*-tests were calculated to test whether the changes from baseline to 3 months, from baseline to 24 weeks and from 12 to 24 weeks were statistically significant. The χ^2^-test was used for evaluation of qualitative variables. Pearson correlations were calculated for the relationship between changes in the metabolic clearance rate of glucose and oxidative stress markers, and changes in volume of visceral fat. Data are presented as mean ± sd and mean with 95% CI.

## Results

Ninety-two per cent of the participants completed the first 12 weeks (95% in the experimental group and 89% in the control group); 84% of the participants in each group completed all 24 weeks. Adherence to the prescribed diet at 24 weeks was high among 55% participants in the experimental group and 32% in the control group, medium among 22.5% in the experimental group and 39% in the control group, and low among 22.5% in the experimental group and 29% in the control group. Pedometer readings and self-reported energy expenditure showed no significant between-group differences. Adherence to the prescribed exercise program was 85.5% (90.3% in the experimental group and 80.6% in the control group).

### Dietary intake

Baseline dietary intake and its changes are shown in Tables 1 and 2. Both groups reduced energy intake (*P* < 0.001 for each group). Percentage of consumed carbohydrates (out of daily caloric intake) increased in the experimental group (*P* = 0.002), with no change in the control group. Percentage of consumed fats decreased in both groups (*P* = 0.03). Percentage of consumed proteins decreased in the experimental group (*P* < 0.001). Cholesterol intake decreased in the experimental group (*P* < 0.001) with no change in the control group.

**Table 2:** Changes in clinical and laboratory variables during the study

	Experimental group		Control group		
			
	Change 3–0	Change 6–0	Change 3–0	Change 6–0	*P*-value
BMI (kg m^−2^)	–2.15 ± 1.42‡	–2.18 ± 2.06‡	–1.21 ± 1.46‡	–0.98 ± 1.57‡	0.001
Total cholesterol (mmol l^−1^)	–0.04 ± 0.74	–0.11 ± 0.81	0.13 ± 0.51	–0.04 ± 0.76	0.730
HDL cholesterol (mmol l^−1^)	–0.05 ± 0.18	–0.01 ± 0.14	0.07 ± 0.16	0.08 ± 0.14*	0.070
LDL cholesterol (mmol l^−1^)	–0.2 ± 0.57*	–0.17 ± 0.68*	–0.16 ± 0.48	–0.14 ± 0.68	0.050
Homocysteine (μmol l^−1^)	–2.9 ± 2.97‡	–4.62 ± 3.4‡	–1.49 ± 3.09*	–3.01 ± 2.92‡	0.170
Triglycerides (mmol l^−1^)	–0.11 ± 0.96	–0.27 ± 0.92	0.03 ± 0.49	0.05 ± 0.63	0.120
Free fatty acids (mmol l^−1^)	–0.09 ± 0.38	0.09 ± 0.66	0.04 ± 0.72	0.24 ± 0.52*	0.140
Fibrinogen	–0.05 ± 1.36	–0.51 ± 1.1*	0.09 ± 0.75	–0.33 ± 0.79*	0.490
Fasting plasma glucose (mmol l^−1^)	–1.45 ± 2.2‡	–1.49 ± 2.03‡	–1.45 ± 2.83†	–1.05 ± 3.2	0.420
Fasting plasma insulin (nmol l^−1^)	–2.85 ± 7.52*	–4.07 ± 9.12‡	–1.92 ± 8.27	–2.93 ± 8.75	0.780
Fasting plasma C-peptide (mIU l^−1^)	–0.1 ± 0.37	–0.18 ± 0.46	–0.21 ± 0.34‡	0 ± 1.08	0.750
HbA_1c_ (DCCT, %) HbA_1c_ (IFCC, mmol/mol)	–0.68 ± 0.86‡ –7 ± 9‡	–0.65 ± 0.99‡ –7 ± 10‡	–0.59 ± 0.89‡ –6 ± 9‡	–0.21 ± 1.1 –2 ± 11	0.370
hsCRP (mg l^−1^)	–1.68 ± 3.61*	–2.27 ± 4.69*	–1.41 ± 4.53	0.09 ± 7.4	0.450
HMW adiponectin (μg ml^−1^)	0.09 ± 1.41	0.59 ± 1.23*	0.54 ± 1.44	0.33 ± 1.49	0.050
Catalase (H_2_O_2_ min^−1^ mg^−1^)	282 ± 290‡	203 ± 237‡	185 ± 237‡	118 ± 224†	0.130
TBARS (μmol l^−1^)	–0.31 ± 0.53†	–0.66 ± 0.54‡	0.27 ± 0.63*	–0.56 ± 0.62‡	0.560
Dietary intake					
Caloric intake (kcal day^−1^)	–137 ± 477‡	–99 ± 438‡	–128 ± 641‡	–36.9 ± 837‡	0.13
Carbohydrates (% of daily energy)	5.07 ± 9.81*	8.24 ± 10.22†	–0.32 ± 9.21	3.15 ± 6.78	0.080
Fats (% of daily energy)	1.95 ± 2.63	–1.22 ± 7.05*	6.2 ± 2.77	–1.33 ± 5.13*	0.610
Proteins (% of daily energy)	–6.87 ± 4.52‡	–4.36 ± 3.05‡	0.52 ± 4.29	–0.23 ± 4.12	< 0.001
Fibre intake (g day^−1^)	6.02 ± 9.58*	2.57 ± 6.62	1.73 ± 9.89	–1.82 ± 8.87	0.030
P/S ratio	0.74 ± 0.55‡	0.57 ± 0.34‡	0.14 ± 0.22*	0.1 ± 0.36	0.004
Cholesterol intake (mg day^−1^)	–322 ± 224‡	–319 ± 206‡	–55.6 ± 187	–22.2 ± 222	< 0.001
Quality of life					
OWLQOL score	5.1 ± 12*	11.5 ± 16‡	6.5 ± 12.7*	7.3 ± 14.9*	0.010
WRSM score	–9.9 ± 14.8‡	–13 ± 20.8‡	–8.1 ± 16.9*	–8.8 ± 17.5*	0.400

Data are means ± sd.

Listed *P*-values are for interaction between group and time assessed by repeated measures ANOVA. *P < 0.05, †*P* < 0.01 and ‡*P* < 0.001 for within-group changes from baseline assessed by paired comparison *t*-tests.

DCCT, Diabetes Control and Complications Trial; H_2_O_2_, hydrogen peroxide; HMW, high molecular weight; hsCRP, high-sensitivity C-reactive protein; IFCC, International Federation of Clinical Chemistry and Laboratory Medicine; OWLQOL, Obesity and Weight-Loss Quality-of-Life; P/S ratio, ratio of polyunsaturated to saturated fatty acids; TBARS, thiobarbituric acid reactive substances; WRSM, Weight-Related Symptoms.

### Glycaemic control, insulin sensitivity

**FIGURE 2:**
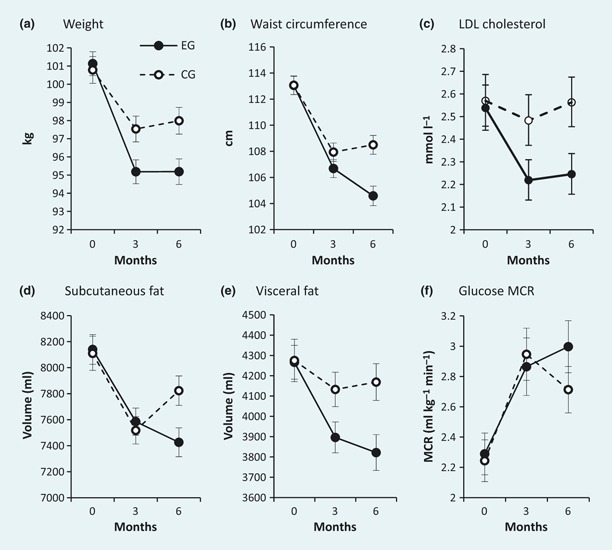
Anthropometric variables, LDL cholesterol, insulin sensitivity, resting energy expenditure and Beck score during the study. Experimental group: closed circles/solid line; control group: open circles/dashed line. Error bars represent 95% CIs. *P*-values for interaction between group and time assessed by repeated measures ANOVA are *P* < 0.001 for weight (a), *P* < 0.001 for waist circumference (b), *P* = 0.05 for LDL cholesterol (c), *P* = 0.02 for subcutaneous fat (d)*, *p* = 0.007 for visceral fat (e)*, *P* = 0.04 for metabolic clearance rate of glucose (MCR) (f)†. *Magnetic resonance imaging data were not complete in 15 out of the 74 participants: 8/37 in the experimental group (six because of dropout, two as a result of claustrophobia during the first examination) and 7/37 in the control group (six because of dropout, one as a result of claustrophobia during the first examination). †Data from the hyperinsulinaemic isoglycaemic clamp were not complete in 19 out of the 74 participants: 9/37 in the experimental group (six because of dropout, three as a result of patients’ fragile veins) and 10/37 in the control group (six because of dropout, four as a result of patients’ fragile veins).

Diabetes medication was reduced in cases of repeated hypoglycaemia in 43% of participants in the experimental group and in 5% of participants in the control group (*P* < 0.001); the difference between groups was 38%(95% CI 17–58%). HbA_1c_ fell in both groups during the first 12 weeks (*P* < 0.001). It remained reduced after exercise. The decrease from baseline to 24 weeks was significant only in the experimental group (–0.65 ± 1%; *P* = 0.002 vs. –0.21 ± 1.1%; NS in the control group), however, the difference between groups was not statistically significant. Among participants whose diabetes medications remained unchanged, HbA_1c_ fell by 0.9% in the experimental group from baseline to 24 weeks (*P* = 0.002) vs. a non-significant decrease of 0.2% in the control group (group × time *P* = 0.08). The metabolic clearance rate of glucose increased in both groups during the first 12 weeks (*P* < 0.001 for each group). After exercise, there were insignificant trends for an increase in the experimental group and a decrease in the control group. The metabolic clearance rate of glucose increased more in the experimental group from baseline to 24 weeks than in the control group [by 30% (95% CI 24.5–39) vs. 20% (95% CI 14–25); group × time *P* = 0.04; Fig. 2f].

### Body weight and abdominal fat

Body weight decreased in both groups in response to the dietary interventions (*P* < 0.001) and it was maintained after the addition of exercise. Weight loss was greater in the experimental group than in the control group [–6.2 kg (95% CI –6.6 to –5.3) vs. –3.2 kg (95% CI –3.7 to –2.5); group × time *P* = 0.001; Fig. 2a]. Waist circumference also decreased in both groups in response to the dietary interventions (*P* < 0.001), more in the experimental group than in the control group [–6.4 cm (95% CI –7.1 to –5.7) vs. –5.3 cm (95% CI –5.9 to –4.5); group × time *P* = 0.001]. After exercise, it further decreased in the experimental group [–1.9 cm (95% CI –2.9 to –1.4); *P* < 0.01], whereas it remained unchanged in the control group [+0.7 cm (95% CI –0.1 to +1.3); NS]. Volume of subcutaneous fat decreased in both groups after the dietary interventions (*P* < 0.001). After the addition of exercise, it further decreased in the experimental group by 2% (95% CI –2.9 to –0.7; *P* < 0.05), whereas it insignificantly increased in the control group by 2% (95% CI –0.1 to +4.2; *P* = 0.06; group × time *P* = 0.02; Fig. 2d). The volume of visceral fat decreased in both groups after the dietary interventions (*P* < 0.001). After the addition of exercise, it further decreased in the experimental group by 4% (95% CI –5.8 to –0.2), whereas it remained unchanged in the control group (group × time *P* = 0.007; Fig. 2e).

### Oxidative stress markers

**FIGURE 3:**
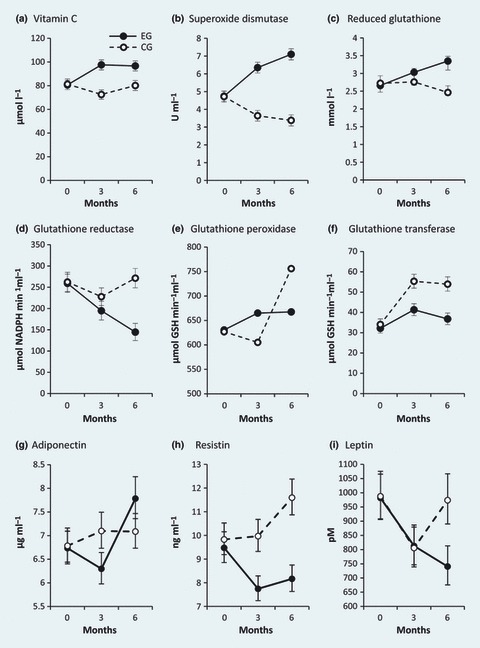
Plasma levels of oxidative stress markers and adipokines during the study. Experimental group: closed circles/solid line; control group: open circles/dashed line. Error bars represent 95% CIs. *P*-values for interaction between group and time assessed by repeated measures ANOVA are *P* = 0.002 for Vitamin C (a), *P* < 0.001 for superoxide dismutase (b), *P* < 0.001 for reduced glutathione (c), *P* < 0.001 for glutathione reductase (d), *P* = 0.004 for glutathione peroxidase (e), *P* = 0.003 for glutathione transferase (f), *P* = 0.02 for adiponectin (g), *P* = 0.005 for resistin (h) and *P* = 0.05 for leptin (i). GSH, glutathione; NADPH, nicotinamide adenine dinucleotide phosphate.

Plasma concentrations of vitamin C increased by 16% (95% CI +13.5 to +24.7) after the diet intervention (*P* = 0.002) and remained elevated after the addition of exercise in the experimental group, whereas changes were not significant in the control group (group × time *P* = 0.002; Fig. 3a). Superoxide dismutase increased in the experimental group in successive steps by 49% (95% CI +44.7 to +57.4; *P* < 0.001), whereas in the control group it gradually decreased by 30% (95% CI –50 to –14; *P* < 0.001; group × time *P* < 0.001; Fig. 3b). Catalase increased in both groups (*P* < 0.01). Thiobarbituric acid reactive substances decreased in both groups (*P* < 0.001). Reduced glutathione increased in the experimental group gradually by 27% (95% CI +16.8 to +29.6; *P* = 0.02; Fig. 3c), whereas it decreased in the control group by 11% (95% CI –19.1 to –0.2; *P* = 0.05; group × time *P* < 0.001). Glutathione reductase decreased in the experimental group gradually by 42% (95% CI –52 to –36; *P* < 0.001; Fig. 3d), while glutathione peroxidase increased in the control group by 20% (95% CI +2 to +44; *P* < 0.001; Fig. 3e) and glutathione transferase increased in both groups, more in the control group than the experimental group [by 59% (95% CI +48.5 to +69.4) vs. 14% (95% CI +6.3 to +24); group × time *P* = 0.003; Fig. 3f].

### Adipokines

Plasma concentrations of both total and high-molecular weight adiponectin increased in the experimental group by 19% (95% CI +7.5 to +25.4) and 15% (95% CI +3.6 to +23.6), respectively, from baseline to 24 weeks, while it did not change significantly in the control group (group × time *P* = 0.02; Fig. 3g, Table 2 and group × time *P* = 0.05, respectively). Resistin decreased after the dietary intervention by 19% (95% CI –25.7 to –10.2) and remained reduced after the addition of exercise in the experimental group, whereas it did not change after the dietary intervention but increased after the addition of exercise by 24% in the control group (95% CI +6.3 to +27.4; *P* = 0.01; group × time *P* = 0.005; Fig. 3h). Leptin decreased similarly in both groups after the dietary interventions, but it increased after the addition of exercise in the control group. A decrease from baseline to 24 weeks was significant only in the experimental group [by 35%; (95% CI –43 to –21.6); *P* = 0.02; group × time *P* = 0.05; Fig. 3i].

### Risk factors of atherosclerosis

LDL cholesterol decreased by 8% after the dietary intervention (95% CI –14 to –5; *P* = 0.05) and remained reduced after exercise in the experimental group, while it did not change in the control group (group × time *P* = 0.05; Fig. 2c). HDL cholesterol increased by 5% from baseline to 24 weeks in the control group (95% CI +3.8 to +9; *P* < 0.01). It increased by 6% in the experimental group after exercise training (95% CI +0.2 to +8.8; *P* = 0.02; group × time *P* = 0.07). Fibrinogen decreased in both groups after exercise training (*P* = 0.02 for the experimental group and *P* = 0.04 for the control group).

### Quality of life

Quality of life increased in both groups, but more in the experimental group (group × time *P* = 0.01).

### Regression analyses, correlations

Regression analyses showed that changes in volume of visceral fat were strongly associated with changes in the metabolic clearance rate of glucose and plasma concentrations of enzymatic oxidative stress markers; each kilogram of lost visceral fat was associated with increases in the metabolic clearance rate of glucose by 1.2 ml kg^−1^ min^−1^, superoxide dismutase by 1.7 U ml^−1^ and reduced glutathione by 0.9 mmol l^−1^. The Pearson’s correlation of metabolic clearance rate of glucose change with change in volume of visceral fat was *r* = –0.63; *P* < 0.001. Correlations between the changes in the volume of visceral fat and both the changes in superoxide dismutase and reduced glutathione were *r* = –0.55; *P* < 0.001 and *r* = –0.45; *P* = 0.02, respectively.

## Discussion

We found that a calorie-restricted vegetarian diet increased insulin sensitivity, reduced volume of visceral fat and improved plasma concentrations of adipokines and oxidative stress markers more than a conventional diet in patients with Type 2 diabetes over 24 weeks. The addition of exercise training further augmented the improved outcomes with our vegetarian diet. To the best of our knowledge, this is the first study that has elucidated the effect of a vegetarian diet and a vegetarian diet plus exercise on these variables.. The advantageous effects of a vegetarian diet may be partly explained by weight loss, especially loss of visceral fat and the consequent increase in insulin sensitivity.

Several possible mechanisms may explain the beneficial effects of a vegetarian diet [17]: higher intake of fibre [18], lower intake of saturated fat [and a higher polyunsaturated and saturated fatty acid (P/S) ratio] [19], higher intake of non-heme iron and reduction in iron stores [20], higher intake of vegetable protein in place of animal protein [21], higher intake of antioxidants [22] and plant sterols [23]. A vegetarian diet was reported to reduce intramyocellular lipid concentrations [24] and this, together with the effect on visceral fat which we observed, might be responsible for a substantial portion of the effect of a vegetarian diet on insulin sensitivity and enzymatic oxidative stress markers.

Our data suggest that a vegetarian diet leads to a complex improvement of enzymatic and non-enzymatic oxidative stress markers. Both enzymatic and non-enzymatic antioxidant defence mechanisms work in synergy against different types of free radicals [25], which play a major role in the development and progression of diabetes and its complications [26]. The changes we observed in plasma concentrations of adipokines reflect loss of adipose tissue.

The reduction in LDL cholesterol observed with our vegetarian diet is in concordance with previous studies where vegetarian diets have been shown to reduce LDL cholesterol [4] and postprandial lipids [27] and to reverse atherosclerosis progression [28]. Of interest is the different dynamics of changes in HDL cholesterol (although the difference between groups was not significant): whereas it increased in the control group from baseline to 6 months with no significant increase in either period, it increased in the experimental group only after the addition of exercise. Previous studies have shown no increase or even a decrease in HDL cholesterol with vegetarian diets; however, this decrease is less than that seen with LDL cholesterol. Isolated increases in HDL cholesterol observed in other diets do not confer the same benefits [29].

The strengths of our study include the parallel design, in which all participants started simultaneously, allowing the investigators to use weekly meetings in both groups to encourage further compliance. Providing all meals for the participants and exercising under professional supervision ensured the best possible compliance. The study duration was long enough to allow sufficient time for adaptation to the diet. The study investigated several metabolic variables, with the results applicable outside of the research setting.

We are aware of several limitations of our study. The number of subjects did not provide sufficient power to confirm the superior effect of the vegetarian diet on HbA_1c_, as observed by Barnard *et al*. [4]. Lower adherence to the prescribed diet in the control group after the addition of exercise points to a potential weakness of the conventional diabetic diet: portion size limits may have increased feelings of hunger during exercise, leading subjects to exceed their prescribed energy intake limits.

The limited adherence to the conventional diabetic diet has been a well-documented problem in dietary intervention studies [4,30]. Especially during exercise, it became evident in our trial that it was easier for subjects to follow a vegetarian diet than a conventional diabetic diet. This may be partly responsible for the greater reduction in volume of visceral fat and insulin resistance with the vegetarian diet after aerobic exercise.

In conclusion, our results indicate that a vegetarian diet alone or in combination with exercise is more effective in increasing insulin sensitivity, reducing volume of visceral fat and improving plasma concentrations of adipokines and oxidative stress markers than a conventional diabetic diet with or without the addition of exercise. Vegetarian diets may provide a beneficial alternative for nutritional therapy in Type 2 diabetes, especially in combination with aerobic exercise. Further studies should explore the precise mechanisms and long-term effects of vegetarian diets in patients with Type 2 diabetes.
